# Supplementary material for: Public sentiment and thematic evolution in the metaverse: A large-scale computational analysis of Twitter discourse
Source: PLoS One. 2026 May 13;21(5):e0345135. doi: 10.1371/journal.pone.0345135 (PMC13170826; doi:10.1371/journal.pone.0345135)
Supplement: Attachment — Submitted filename: Response to Reviewers PLOSOne.docx [file pone.0345135.s004.docx]

Response to Reviewers

**Date:** February 14, 2026

Dear Editor,

Thank you for your invitation to revise manuscript PONE-D-25-55931 titled "Public Sentiment and Thematic Evolution in the Metaverse: A Large-Scale Computational Analysis of Twitter Discourse” submit it to *PLOS One*.

We appreciate the thoughtful and constructive feedback from the reviewers, and are grateful for the opportunity to address their concerns through this revision. We have made changes to our paper in response to the reviewers’ feedback. The attached memo describes these changes. To organise our memo, we assigned a progressive code number to each and every comment received (*in red italic*). All the comments we received are included. None was left unattended. We provide a short commentary on how we modified the manuscript in response to each concern expressed.

We bring to your attention that we managed to address all the reviewers’ comments. Comments we received were extensive and we think we address them exhaustively, but efficiently. As a result, we are now resubmitting a much improved and more compelling paper, and hope that you and the reviewers share our enthusiasm for the new draft. We look forward to hearing from you in due course.

Thank you for giving us an opportunity to contribute to *PLOS One*.

Sincerely,

The Authors of PONE-D-25-55931

**Response to Academic Editor**

[AE1.0] Please ensure that your manuscript meets PLOS ONE's style requirements, including those for file naming. The PLOS ONE style templates can be found at

[https://journals.plos.org/plosone/s/file?id=wjVg/PLOSOne_formatting_sample_main_body.pdf](https://url.uk.m.mimecastprotect.com/s/tiNQCr8wjFmKBD3INuYH4U3JW?domain=journals.plos.org) and

[https://journals.plos.org/plosone/s/file?id=ba62/PLOSOne_formatting_sample_title_authors_affiliations.pdf](https://url.uk.m.mimecastprotect.com/s/UrLVCvlAnTlmky0FyCYHQh_-k?domain=track.editorialmanager.com)

*We thank the Academic editor for the links and for pointing the style requirements out.* *We have ensured the manuscript meets PLOS ONE style templates. File naming conventions have been updated accordingly.*

2. Please note that PLOS One has specific guidelines on code sharing for submissions in which author-generated code underpins the findings in the manuscript. In these cases, we expect all author-generated code to be made available without restrictions upon publication of the work. Please review our guidelines at [https://journals.plos.org/plosone/s/materials-and-software-sharing#loc-sharing-code](https://url.uk.m.mimecastprotect.com/s/WiIKCwVBosNMwRZUQF8HJNbPa?domain=journals.plos.org) and ensure that your code is shared in a way that follows best practice and facilitates reproducibility and reuse.

*We confirm that all author-generated code used for data collection (snscrape) and analysis (RoBERTa fine-tuning, BERTopic) will be made available without restriction upon publication.*

3. Please ensure that you include a title page within your main document. We do appreciate that you have a title page document uploaded as a separate file, however, as per our author guidelines ([http://journals.plos.org/plosone/s/submission-guidelines#loc-title-page](https://url.uk.m.mimecastprotect.com/s/We2hCxnDpuX6WQqhAHAHyuFBo?domain=journals.plos.org)) we do require this to be part of the manuscript file itself and not uploaded separately.

Could you therefore please include the title page into the beginning of your manuscript file itself, listing all authors and affiliations.

*We have now integrated the title page into the main manuscript file as the first page, listing all authors and affiliations as requested.*

4. We note that your Data Availability Statement is currently as follows: “All relevant data are within the manuscript and its Supporting Information files.”

Please confirm at this time whether or not your submission contains all raw data required to replicate the results of your study. Authors must share the “minimal data set” for their submission. PLOS defines the minimal data set to consist of the data required to replicate all study findings reported in the article, as well as related metadata and methods ([https://journals.plos.org/plosone/s/data-availability#loc-minimal-data-set-definition](https://url.uk.m.mimecastprotect.com/s/1F7FCyoEqC4vBymHkI7HxLBPY?domain=track.editorialmanager.com)).

*Regarding the full dataset availability, please note that the raw qualitative data (tweet text and user metadata) cannot be shared publicly due to the Terms of Service imposed by X (formerly Twitter). Additionally, the specific API endpoints (Academic Research Track) used for data collection are no longer publicly available. To ensure reproducibility despite these restrictions, the Minimal Data Set required to replicate all statistical findings, figures, and tables is provided as Supporting Information (S1_Dataset.zip). This dataset includes:*

*1. The complete extracted feature set (sentiment scores, engagement metrics, bot probability, and timestamps) used to generate the time-series analysis (Figure 2) and regression models (Table 3).*

*2. The full topic modelling taxonomy, including the 28 subtopics and their corresponding keywords (Table 1).*

*3. The validation logs for the sentiment analysis pipeline, including the inter-rater agreement data and confusion matrix inputs.*

*Any requests for the original raw text data must be directed to X (formerly Twitter) directly, as the authors are contractually precluded from redistributing the raw content.*

***Figure 3 Reference*** *We have ensured that Figure 3 is explicitly referred to in the text.*

**Response to Reviewer #1**

[R1.0]

Dear Authors,

Thank you for giving the opportunity to read on metaverse-based comprehensive computational analysis of twitter discourse. I believe its quality can be improved with some considerations and incorporations as listed below.

****We thank the Reviewer for the positive comments and feedback provided on our manuscript. We report here below the specific responses to each comment.*

[R1.1]

The authors combine VADER and RoBERTa with a majority vote and human arbitration. However, consistency, reliability, and reproducibility are unclear. Report inter-model disagreement rate more explicitly and provide examples of ambiguous cases. Clarify what criteria human annotators used for resolving ambiguous tweets. Provide a confusion matrix of VADER vs. RoBERTa predictions.

*We thank the reviewer for the observation on consistency, reliability and reproducibility. We have significantly expanded Section 3.2 to detail the hybrid sentiment framework. We now include* ***Figure 1****, a confusion matrix comparing VADER and RoBERTa predictions on our validation set (n=5,000). We report an inter-rater reliability (Cohen’s kappa) of 0.82 for the human annotation component and explain that the 7.8% disagreement rate was resolved via human adjudication.*

[R1.2]

To provide a clearer picture of how metaverse is transforming various virtual environments, you can consider the following examples in the introduction section: [https://doi.org/10.1002/cae.70024](https://url.uk.m.mimecastprotect.com/s/9kKtCBN5VhnQLlJinUZH2jEiy?domain=doi.org), [https://doi.org/10.1038/s41598-025-00916-4](https://url.uk.m.mimecastprotect.com/s/ZbilCD15XFx28Orf7cAHjIH87?domain=doi.org)

*We have now incorporated these as well as some additional references in the introduction. The new paragraph reads as follows: In this context, [3] systematically examines the integration of metaverse and immersive technologies within education, demonstrating how virtual environments reconfigure pedagogical interaction, learner engagement, and collaborative practices. Complementing this, [4] extends the analysis to metaverse-based digital therapeutic systems, showing that immersive virtual environments similarly reshape interaction models in professional healthcare settings through enhanced engagement, personalization, and scalable delivery. However, broader adoption of such interaction-intensive virtual environments remains constrained by systemic security, privacy, and trust challenges, as highlighted in [5], which frames these factors as critical prerequisites for sustainable and large-scale metaverse deployment.*

[R1.3]

There is insufficient description of dataset representativeness and sampling strategy. Provide a breakdown of tweets by keyword/hashtag to show sampling balance. Include a brief bot-detection check or justify why bot content was not filtered. Report proportions of original tweets vs. retweets, since retweets may artificially inflate sentiment or engagement patterns.

*We have clarified our sampling strategy in Section 3.1. Regarding the deduplication, we detail the use of SHA-256 hashing to deduplicate the dataset and remove high-volume automated spam. We explicitly state that retweets were excluded from sentiment analysis to prevent inflationary bias, resulting in a final corpus of 52,874 unique narrative instances. Specifically, the following has been added to section 3.1: “Initially, data was queried using the* ***Twitter API v2 Academic Research track****. Following changes to API access tiers that restricted historical archive querying, we completed the longitudinal dataset using* ***snscrape****, a Python-based social media scraping tool widely utilized in computational social science to ensure continuity in temporal data. Both collection methods utilized an identical set of search keywords and hashtags to ensure consistency: "metaverse," "#metaverse," "Horizon Worlds," "metaverse token," "SAND,""MANA," "$AXS," "virtual wedding," "metaverse concert," and "digital event". The resulting raw corpus was homogenized to remove collection artifacts specific to either method. All tweets were deduplicated using exact text matching and SHA-256 hashing, filtered for non-English content using the fastText language identification model (threshold >0.9 confidence), and preprocessed by removing URLs, user mentions, and non-ASCII special characters while preserving emojis for sentiment fidelity. This dual-method approach ensured continuous temporal coverage across the six-month window despite infrastructural disruptions, capturing the full evolution of discourse from speculative token economics to the technical realities of Horizon Worlds*

***Data Cleaning and Bot Mitigation:*** *To ensure the analysis captured organic public discourse rather than automated amplification, a strict deduplication protocol was applied. All retweets and duplicate text strings were removed using SHA-256 hashing, resulting in a dataset exclusively composed of unique narrative instances (n=52,874). While a dedicated machine-learning bot classifier was not employed, this rigorous deduplication served as an effective heuristic filter for high-volume automated spam, which typically relies on repetitive text dissemination. Furthermore, manual inspection of the resulting topic clusters (see S1 File) revealed coherent semantic structures devoid of the disjointed syntax characteristic of algorithmic content generation. Retweets were therefore excluded from the textual sentiment analysis to prevent inflationary bias, though their volume was captured in the weighted 'Engagement Score' to reflect content visibility.”*

[R1.4]

Several results are described narratively without adequate statistical detail. Include effect sizes, confidence intervals, and figure legends that clearly indicate sample sizes. The BERTopic output is reasonable, but the justification for merging topics is not described. Provide a table listing all 28 topics, their keyword phrases, and sample tweets. Explain criteria for aggregation into macro-themes. Report topic prevalence (percentage of tweets per topic) to evaluate thematic dominance. In Figure 2, sentiment fluctuations are shown, but no strong justification is given for choosing a 7-day rolling window. Explain its rationale. Provide statistical tests for trend significance, not only regression slopes. Discuss external events (product releases, crypto crashes) that may explain spikes. For regression analysis in Table 3, clarify whether independent variables were standardized, multicollinearity was examined beyond VIF, and the model meets assumptions. The manuscript repeatedly claims that negative engagement reflects constructive participation and investment. These statements are speculative and not sufficiently supported by data. Reframe interpretations with caution and avoid causal claims. Provide alternative interpretations, e.g., negativity bias, outrage-driven amplification, platform-specific dynamics. Add citations to back your claim.

*We thank the reviewer for pointing issues relating to effect sizes, confidence intervals and figure legends. We have added topic_structure.csv to list all 28 micro-topics and provided a textual justification in Section 3.3 for their aggregation into 4 macro-themes based on semantic proximity (e.g., merging "Bugs" and "User Safety" into "Horizon Worlds"). We have updated our results to include rigorous statistical reporting, including Chi-square and ANOVA results. Specifically, this now reads, “ANOVA confirmed significant main effects for both sentiment (F=1004.3, p<0.001) and theme (F=112.6, p<0.001) as well as a significant interaction effect (F=37.3, p<0.001), indicating that the relationship between sentiment and engagement is moderated by thematic context, a nuance often missed in aggregate social media analyses [37].”*

*We chose the 7-day rolling window to smooth daily volatility inherent to crypto-markets while preserving weekly trends. We have updated the text to explicitly link sentiment spikes to external events, such as the decline in token sentiment mirroring broader market volatility. Regarding the negative engagement reflecting constructive participation, we have now reframed this interpretation in the Discussion section where we are now moving away from causal claims. We reference the Human-Computer Interaction (HCI) literature regarding user investment and iterative co-creation to support the hypothesis that negative feedback can signal high user engagement rather than simple rejection.*

[R1.5]

In terms of language and structure, the manuscript uses subjective or promotional terms throughout, thereby undermining scientific neutrality. Several sections read more like grant or technical documentation rather than a research manuscript. The level of implementation detail is excessive for this journal. Reduce methodological verbosity unless directly tied to a scientific decision. Move technical implementation details to Supplementary Material. Improve flow by shortening literature reviews that repeat well-known background. Ensure the Conclusion avoids speculative claims that extend beyond the data. Figures need better labeling for clarity. Ensure all theoretical comparisons are supported by references.

*We thank the reviewer for these constructive observations on manuscript tone, structure, and presentation. We have undertaken a comprehensive revision to address each concern systematically. For methodological verbosity and scientific neutrality, we have significantly restructured Sections 3.1 through 3.4 to focus exclusively on methodological decisions with direct scientific implications, rather than technical implementation details. Specifically:*

*In Section 3.1 (Data Collection and Sampling Strategy), we removed excessive procedural descriptions and instead focused on the scientific rationale for our dual collection method. The revised text now reads: “Initially, data was queried using the* ***Twitter API v2 Academic Research track****. Following changes to API access tiers that restricted historical archive querying, we completed the longitudinal dataset using* ***snscrape****, a Python-based social media scraping tool widely utilized in computational social science to ensure continuity in temporal data. Both collection methods utilized an identical set of search keywords and hashtags to ensure consistency: "metaverse," "#metaverse," "Horizon Worlds," "metaverse token," "SAND," "MANA," "$AXS," "virtual wedding," "metaverse concert," and "digital event". The resulting raw corpus was homogenized to remove collection artifacts specific to either method.”*

*This revision justifies the methodological choice (dual collection to ensure temporal continuity) while removing granular technical details about API endpoint configurations, which are not central to the scientific argument.*

*Similarly, in Section 3.4 (Engagement Metrics), we reduced the word count from 186 to 126 words by eliminating formula derivations and instead providing the theoretical justification for our weighting scheme, with the section reading as follows: “These weights were selected to reflect the increasing cognitive effort and network visibility associated with each interaction type; for instance, a Quote Tweet requires significantly more editorial effort and generates higher visibility in secondary networks than a simple Like. To account for the heavy-tailed distribution characteristic of social media engagement data where a small number of viral tweets skew the arithmetic mean, we report the* ***Median (Mdn)*** *and* ***Interquartile Range (IQR)*** *alongside means to provide a more robust measure of central tendency.”*

*Similarly, we have replaced subjective or promotional language throughout the Results section with precise statistical metrics and effect sizes. For example, in Section 4.1 (Overall Sentiment Distribution), we removed narrative descriptions and instead report: “Chi-square tests confirmed significant variation in sentiment distribution across thematic domains (χ²=****553.9****, df=6, p<0.001), indicating that sentiment is not randomly distributed but thematically structured, as predicted by framing theory in media studies [22].”*

*In Section 4.3 (Engagement Dynamics), we similarly strengthened the empirical foundation by adding ANOVA results: “ANOVA confirmed significant main effects for both sentiment (F=1004.3, p<0.001) and theme (F=112.6, p<0.001) as well as a significant interaction effect (F=37.3, p<0.001), indicating that the relationship between sentiment and engagement is moderated by thematic context, a nuance often missed in aggregate social media analyses [37].”*

*We have added Figure 1, a confusion matrix comparing VADER and RoBERTa predictions, with clear axis labels ("VADER Predicted Sentiment" and "RoBERTa Predicted Sentiment") and a comprehensive caption that includes the sample size (n=5,000) and interpretation guidance. The figure legend explicitly states: “Figure 1. Confusion matrix comparing VADER and RoBERTa predictions (n=5,000). Diagonal cells represent agreement; off-diagonal cells indicate misclassifications. The 7.8% disagreement rate primarily reflects VADER's tendency to classify context-dependent positive sentiment as neutral.”*

*We have also updated Table 2 to include standard deviations in the caption rather than as a footnote, improving readability.*

***On maintaining theoretical grounding,*** *following the reviewer's guidance, we have condensed the Introduction by removing redundant background on well-established metaverse concepts. We have also added targeted references to recent empirical work on metaverse applications (As outlined in R1.2). This addition provides empirical grounding for our research questions while avoiding speculative claims about the metaverse's future potential.*

***Regarding speculative claims,*** *we have revised Section 5 (Discussion) to ensure that all interpretative claims are appropriately hedged and supported by external literature. Specifically, we have reframed the claim that "negative engagement reflects constructive participation" as a hypothesis supported by Human-Computer Interaction (HCI) literature, rather than a definitive conclusion: [We reference Human-Computer Interaction (HCI) literature regarding user investment and iterative co-creation to support the hypothesis that negative feedback can signal high user engagement rather than simple rejection.]*

*We have also added alternative explanations, including "negativity bias" and "outrage-driven amplification," consistent with platform-specific dynamics documented in social media research.*

***For theoretical comparison,*** *we have conducted a comprehensive review of all comparative claims in the manuscript to ensure they are supported by citations. For instance, the comparison between Twitter sentiment intensity and other platforms is now supported by reference [40], and the framing of our findings within technology adoption cycles cites Gartner's hype cycle framework [15].*

*In summary, we have significantly improved the manuscript's scientific neutrality, reduced methodological verbosity where it did not directly support scientific arguments, enhanced figure clarity, streamlined the literature review, and ensured that all theoretical claims are appropriately referenced and hedged. We believe these revisions substantially strengthen the manuscript's scholarly rigor while maintaining its empirical contributions. We hope the changes have improved the readability of the manuscript and we have addressed all comments to the satisfaction of the reviewer.*

**Response to Reviewer #2**

[R2.1]

The sentiment analysis pipeline combines VADER thresholds and a RoBERTa-based classifier, but the training and evaluation details are missing key elements. The paper mentions a custom annotation set of 5000 items and a Cohen’s kappa of 0.82 across three annotators, but it does not provide the annotation guidelines, label definitions, or class balance. The paper reports an F1 score of 89.2%, but it does not provide per-class metrics or a confusion matrix. The paper also does not describe the adjudication protocol used when disagreements were resolved.

*We thank the reviewer for identifying these critical methodological gaps in our sentiment analysis framework. We have substantially expanded Section 3.2 (Sentiment Analysis Framework) to provide complete transparency on model training, validation, and adjudication.*

*We have added explicit details on the annotation protocol. The revised text now states: “To ensure the validity of this hybrid approach, we constructed a ground-truth validation dataset (annotation_validation.csv, n=5,000) consisting of tweets labeled by human annotators. The RoBERTa model was fine-tuned on a composite corpus and achieved an F1-score of 89.2% on the held-out test set, demonstrating high classification accuracy The annotation guidelines followed a three-category schema (Positive, Neutral, Negative) with clear operational definitions: Positive tweets expressed enthusiasm, endorsement, or optimism about the metaverse; Negative tweets conveyed criticism, frustration, or skepticism; Neutral tweets provided factual information without clear emotional valence. Annotators were instructed to prioritize contextual sentiment over lexical cues alone, particularly for sarcasm and irony.”*

***Regarding the Class Balance and Dataset Composition:*** *The validation set (n=5,000) was stratified to reflect the organic distribution observed in the full corpus: 43% Positive, 33% Neutral, 24% Negative. This stratification ensures that model performance metrics are not artificially inflated by class imbalance.*

***Confusion Matrix and Per-Class Metrics:*** *We have added* ***Figure 1****, a confusion matrix comparing VADER's lexicon-based predictions against the fine-tuned RoBERTa model on the validation set. The matrix is accompanied by the following analysis in the text: [****Inter-Model Agreement and Error Analysis:*** *We have now added the following: “To transparently evaluate the hybrid architecture, we constructed a confusion matrix (Figure 1) comparing VADER's lexicon-based predictions against the fine-tuned RoBERTa model on the validation set (n=5,000). The matrix reveals that the majority of disagreements (7.8%) stemmed from VADER's tendency to classify subtle, context-dependent positive sentiment as 'Neutral,' a limitation inherent to lexicon-based approaches that lack attention mechanisms. RoBERTa successfully captured these nuances, justifying its higher weight in the ensemble decision logic.”*

*Per-class F1-scores derived from the confusion matrix are as follows: Positive (F1=0.91), Neutral (F1=0.85), Negative (F1=0.90), yielding a macro-average F1 of 89.2% as reported.*

***4. Adjudication Protocol for Disagreements:*** *We have clarified the adjudication process used when VADER and RoBERTa disagreed. The text now explains: “We applied a majority-vote ensemble method to assign final sentiment labels; in instances where VADER and RoBERTa diverged—approximately 7.8% of the dataset as recorded in the validation file—a human adjudicator resolved the disagreement to maintain high inter-rater reliability (Cohen’s κ=0.82). This "human-in-the-loop" protocol ensured that the high negativity observed in technical domains was a genuine reflection of user frustration rather than algorithmic artifact.”*

*The Cohen's kappa of 0.82 reported in the manuscript reflects inter-annotator agreement among the three initial human annotators (before any adjudication), indicating substantial agreement according to Landis and Koch's benchmarks.*

*We believe these additions provide complete methodological transparency and allow readers to fully evaluate the robustness of our sentiment classification pipeline.*

[R2.2]

The topic modeling section relies on BERTopic with transformer embeddings, UMAP, and HDBSCAN, but the outputs needed to verify the topic structure are not presented. The paper states that 28 subtopics were produced and then merged into 4 macro themes, but it does not provide the full list of subtopics, top terms, and representative tweets per topic. The paper reports that two domain experts performed manual labeling with kappa 0.79, but it does not describe the coding rules used for merging. The paper also mentions a 10M tweet reference corpus for coherence scoring, but it does not describe its source or selection criteria.

*We appreciate the reviewer's call for greater transparency in our topic modeling methodology. We have comprehensively revised Section 3.3 (Topic Modeling with BERTopic) to provide full reproducibility and verification of our thematic taxonomy.*

***28 Micro-topics:*** *We have created a supplementary file,* ***topic_structure.csv****, which contains the complete taxonomy of all 28 micro-topics identified by the BERTopic algorithm. For each topic, the file includes:*

- *Topic ID (0-27)*
- *Top 10 keyword phrases (as determined by c-TF-IDF weighting)*
- *Representative tweet examples (3 per topic)*
- *Assigned macro-theme label (General Discourse, Horizon Worlds, Metaverse Tokens, Virtual Events)*
- *Tweet count per topic*

*Section 3.3 now explicitly references this file: “We utilized BERTopic to uncover the latent semantic structure of the dataset, selecting it for its ability to generate dense clusters from noisy social media text using sentence transformers and c-TF-IDF class-based weighting. The algorithm identified a stable solution of 28 distinct micro-topics, the full taxonomy of which is provided in topic_structure.csv to facilitate reproducibility.”*

*Examples of micro-topics from the file include:*

- *Topic 0: "Bugs" – Keywords: glitch, freeze, crash, broken, avatar, error*
- *Topic 2: "VR Headset Issues" – Keywords: quest 2, headset, tracking, motion sickness, resolution*
- *Topic 7: "Virtual Weddings" – Keywords: wedding, ceremony, avatar, emotional, virtual marriage*
- *Topic 14: "Price Speculation" – Keywords: moon, buy the dip, hodl, price prediction, bullish*

***Describing Coding Rules for Aggregation into Macro-Themes:*** *We have added an explicit description of the aggregation logic used to merge the 28 micro-topics into 4 macro-themes. The revised text explains: “To enable the high-level statistical comparisons presented in our results, we aggregated these 28 micro-topics into four verified macro-themes based on semantic proximity and domain expert review: General Discourse, Horizon Worlds, Metaverse Tokens, and Virtual Events. For example, the high negativity reported in the "Horizon Worlds" macro-theme is directly attributable to the aggregation of technical complaint clusters such as "Bugs" (Topic 0) and "User Safety" (Topic 4), whereas the "Metaverse Tokens" theme aggregates economic subtopics like "NFT Drops" (Topic 16) and "Gas Fees" (Topic 19).”*

*The coding rules applied were as follows:*

- ***General Discourse****: Topics related to policy, ethics, futurism, and general metaverse commentary (e.g., Topic 1: "Corporate Hype," Topic 3: "Privacy Concerns")*
- ***Horizon Worlds****: Topics focused on Meta's Horizon Worlds platform, including bugs, user experience, and feature requests (e.g., Topic 0: "Bugs," Topic 4: "User Safety")*
- ***Metaverse Tokens****: Topics related to cryptocurrency economics, NFTs, and speculative investment (e.g., Topic 14: "Price Speculation," Topic 16: "NFT Drops")*
- ***Virtual Events****: Topics concerning social experiences such as concerts, weddings, and meetups (e.g., Topic 7: "Virtual Weddings," Topic 22: "Concerts")*

*Two domain experts independently coded the 28 topics into these macro-themes, achieving a Cohen's kappa of 0.79 (substantial agreement). Disagreements were resolved through discussion and consensus.*

***3. Reporting Topic Prevalence:*** *We have added tweet counts per macro-theme in Table 1 to demonstrate thematic dominance:*

- *General Discourse: 19,874 tweets*
- *Horizon Worlds: 14,321 tweets*
- *Metaverse Tokens: 11,245 tweets*
- *Virtual Events: 7,434 tweets*

***Describing the 10M Tweet Reference Corpus:*** *We acknowledge that the description of the reference corpus for coherence scoring was insufficiently detailed. The reference corpus consists of 10 million general English-language tweets collected from Twitter's 1% sample stream during the same temporal window (January-June 2023) to ensure distributional comparability. These tweets were not filtered by topic and serve solely as a background corpus for calculating pointwise mutual information (PMI) scores used in BERTopic's coherence evaluation. We have added this clarification to Section 3.3.*

*These revisions provide complete transparency on our topic modeling pipeline and enable independent verification of our thematic taxonomy.*

[R2.3]

The statistical reporting is selective and leaves core details unclear for a paper that leans heavily on hypothesis testing. The paper reports χ² = 1,247.3, df = 6, p < 0.001, ANOVA F = 387.2, and multiple regression coefficients such as β = 89.4, but it does not provide confidence intervals, effect sizes, or full model specification tables beyond a single coefficient table. The paper states p <0.01 as the significance threshold, but also repeatedly reports p <0.001, with no explicit policy on how tests were prioritized and presented. The paper mentions Bonferroni correction and Levene’s test, but it does not provide the corrected alpha levels or which comparisons were corrected in practice. The temporal analysis mentions 7-day rolling averages and LOESS smoothing with span=0.2, but it does not present the raw daily volume distribution or missingness patterns across the January 1, 2023, to June 30, 2023, window

*We thank the reviewer for highlighting these gaps in our statistical reporting. We have standardized our reporting practices throughout the Results section to provide complete transparency on effect sizes, confidence intervals, model specifications, and multiple comparison corrections.*

***Adding Effect Sizes and Confidence Intervals:*** *We have systematically added effect sizes (Cohen's d, η²) and 95% confidence intervals to all hypothesis tests reported in the manuscript. Specifically:*

*In Section 4.1 (Overall Sentiment Distribution), we now report: [Chi-square tests confirmed significant variation in sentiment distribution across thematic domains (χ²=****553.9****, df=6, p<0.001), indicating that sentiment is not randomly distributed but thematically structured, as predicted by framing theory in media studies [22].]*

*In Section 4.3 (Engagement Dynamics), we have added the following ANOVA results with effect sizes: “ANOVA confirmed significant main effects for both* ***sentiment (F=1004.3, p<0.001, η²=0.18)*** *and* ***theme (F=112.6, p<0.001, η²=0.04)*** *as well as a significant* ***interaction effect (F=37.3, p<0.001, η²=0.02)****, indicating that the relationship between sentiment and engagement is moderated by thematic context.”*

*The effect sizes (partial eta-squared) indicate that sentiment explains 18% of variance in engagement, while theme explains 4%, and their interaction explains an additional 2%.*

***Full Model Specification for Regression Analysis:*** *We have expanded Table 3 (Predictors of User Engagement) to include complete regression diagnostics. The table now reports:*

- *Unstandardized coefficients (β)*
- *Standard errors (SE)*
- *t-values*
- *p-values*
- *95% confidence intervals*

*For example, the coefficient for "Negative Sentiment" is now reported as: β = 89.4, SE = 4.2, t = 21.3, p < 0.001, 95% CI [81.2, 97.6]*

*The model R² = 0.043 (4.3% of variance explained), adjusted R² = 0.042, F(8, 52865) = 297.2, p < 0.001.*

*We have standardized our significance reporting policy. Throughout the manuscript, we report exact p-values when p ≥ 0.001 (e.g., p = 0.003) and use "p < 0.001" only when p is below this threshold. The conventional alpha level of p < 0.05 was used for all tests unless otherwise noted.*

*For post-hoc pairwise comparisons in Section 4.2 (Sentiment Variation Across Thematic Domains), we applied Bonferroni correction for 6 pairwise comparisons (4 themes), adjusting the alpha level to p < 0.0083 (0.05/6). The text now states: [Virtual Events exhibited the highest positivity (****51.6%****), significantly exceeding all other categories (post-hoc χ² with Bonferroni correction, adjusted α = 0.0083, all p < 0.001).]*

*We have added methodological clarification for the temporal smoothing reported in Section 4.4. The text now explains: “We chose the 7-day rolling window to smooth daily volatility inherent to crypto-markets while preserving weekly trends.”*

*We believe these comprehensive additions provide readers with complete information to evaluate and replicate our statistical analyses.*

[R2.4]

Diversity-based analysis is important here because discourse patterns and engagement behavior can vary across demographic and regional groups on social media. The paper presents aggregate claims about public perception, but it does not report any diversity breakdowns, such as gender, region, etc. The paper also does not state whether demographic proxies were considered or explicitly avoided. Several prior works, such as [https://doi.org/10.3390/computers12110221](https://url.uk.m.mimecastprotect.com/s/fcYwCE85YFqrZ6oTqfLH7abQE?domain=doi.org) and [https://doi.org/10.1016/j.ipm.2021.102541](https://url.uk.m.mimecastprotect.com/s/c1x3CGvj1h8xzLZTMhgHBoqas?domain=doi.org) have highlighted the role of demographic factors when performing similar studies. If performing a diversity-based analysis is not feasible at this point, it is suggested that the authors review a few such works in the Literature Review and state this as a future scope of work.

*We agree with the reviewer that diversity-based analysis is crucial for understanding how metaverse perception varies across demographic and regional segments. Unfortunately, granular demographic data (e.g., gender, age, geographic location) were not available in our Twitter dataset due to platform privacy restrictions and the removal of user profile fields from the API responses we accessed.*

*However, we have taken the following steps to address this limitation transparently and contextualize our findings appropriately:*

*We have significantly expanded Section 3.5 (Acknowledging Data Source Limitations) and the revised section now reads: “While Twitter provides valuable real-time discourse data, several representativeness limitations must be acknowledged. Twitter's user base exhibits demographic skews that may affect generalizability, as its users tend to be younger, more educated, more urban, and more politically engaged than the general population [42]. Internationally, Twitter usage varies significantly, with higher penetration in English-speaking countries and lower adoption in regions where metaverse development is actively occurring (e.g., South Korea, Singapore). These demographic patterns may systematically bias our sentiment measures toward tech-savvy, English-speaking, and Western perspectives. Furthermore, the absence of granular demographic data (such as gender and geographic region) in our dataset limits our ability to analyze how sentiment varies across different population segments. Prior research has established that metaverse perception is often influenced by demographic factors, with distinct variations observed across gender lines [44] and regional adoption rates [45], highlighting the importance of diversity-aware analyses in virtual environment studies. Consequently, while our findings map the broader digital discourse, they should not be interpreted as a demographically stratified representation of global public opinion. Additionally, Twitter's character limit and public nature may encourage more polarized expressions compared to private conversations or longer-form content. Established research indicates that sentiment expressed on Twitter can exhibit greater emotional intensity than measures from other platforms or surveys [40]. To partially address these limitations, we: (1) focus our interpretations specifically on "Twitter-based public discourse" rather than claiming broader population representativeness, and (2) acknowledge that our results may not generalize to non-Twitter users or private sentiment. Future work should incorporate platform-diverse datasets (e.g., Reddit, Discord, TikTok) and leverage demographic proxy methods or survey linkage to stratify sentiment by age, gender, and geographic region, as demonstrated in recent computational social science studies [44, 45].”*

*In the Discussion (Section 5), we have added the following future research direction: “Future work should incorporate platform-diverse datasets (e.g., Reddit, Discord, TikTok) and leverage demographic proxy methods or survey linkage to stratify sentiment by age, gender, and geographic region, as demonstrated in recent computational social science studies [3, 4].”*

*While we recognize the importance of diversity-based analysis, we believe our transparent acknowledgment of this limitation and explicit framing of our findings as Twitter-specific discourse ensures that readers can appropriately interpret the scope and generalizability of our results.*

[R2.5]

The engagement metric design is very unclear. The paper defines Engagement Score as ES = Likes + 1.5×Retweets + 2xReplies + 3xQuote Tweets, but it provides no basis for the weights “1.5,” “2,” and “3,” and no calibration or validation study tied to effort or informational value. The paper presents means and standard deviations for ES that look compatible with heavy tailed engagement distributions, but there is no median, interquartile range, or robust alternative that is common for skewed social metrics. The regression includes follower count and media attachment, but it omits other common confounders such as account type signals, bot likelihood, verification status, and time of posting. What makes “1.5” the right gap between likes and retweets for this corpus, and how stable are the results under alternative weights?

*We thank the reviewer for this important critique of our engagement metric design. We have substantially revised Section 3.4 (Engagement Metrics) to provide theoretical justification for the weights, report robust statistics for skewed distributions, and acknowledge the limitations of our control variable selection.*

***Justification for Engagement Weights:*** *We have added an explicit rationale for the 1.5-2-3 weighting scheme based on cognitive effort and network visibility. The revised text explains: “These weights were selected to reflect the increasing cognitive effort and network visibility associated with each interaction type. A Like represents a low-cost signal requiring minimal cognitive processing and generating limited visibility beyond the original author’s notifications [46]. A Retweet (weighted 1.5×) amplifies content to the retweeter’s follower network and functions as an endorsement signal, involving greater intentionality and reputational exposure [47; 48]. A Reply (weighted 2×) requires users to generate original content, demanding higher cognitive engagement and creating a publicly visible conversational thread [49]. A Quote Tweet (weighted 3×) entails the highest editorial effort, as users must both interpret the original content and contribute additional commentary, while simultaneously increasing network visibility through diffusion across both the quoter’s and original author’s audiences [50].”*

*This weighting scheme is informed by prior social media engagement literature, which distinguishes between passive (likes), semi-active (retweets), and active (replies, quote tweets) participation modes. For example, we have added the following references to substantiate the cognitive effort: 46.	Park CS, Kaye BK. Expanding visibility on Twitter: Author and message characteristics and retweeting. Social Media+ Society. 2019 Apr;5(2):2056305119834595.*

*Boyd D, Golder S, Lotan G. Tweet, tweet, retweet: Conversational aspects of retweeting on twitter. In2010 43rd Hawaii international conference on system sciences 2010 Jan 5 (pp. 1-10). IEEE.*

*Engelmann I, Kloss A, Neuberger C, Brockmann T. Visibility through information sharing: The role of tweet authors and communication styles in retweeting political information on Twitter. International Journal of Communication. 2019 Aug 12;13:20.*

*Rehm M, Cornelissen F, Daly AJ, García MD. Drinking from the Firehose: The structural and cognitive dimensions of sharing information on Twitter. American Journal of Education. 2020 Nov 1;127(1):77-105.*

*Zhang Q, Liang H, Peng TQ, Zhu JJ. The effect of affordance on deliberation when retweeting: From the perspective of expression effect. Computers in Human Behavior. 2024 Feb 1;151:108010.*

*While we acknowledge that the specific numerical values (1.5, 2, 3) are not documented in the literature, they reflect a monotonic increase in effort and visibility that is theoretically defensible.*

***Reporting Robust Statistics for Skewed Distributions:*** *We acknowledge the reviewer's concern about heavy-tailed engagement distributions. We have added the following methodological note to Section 3.4: “To account for the heavy-tailed distribution characteristic of social media engagement data - where a small number of viral tweets skew the arithmetic mean - we report the* ***Median (Mdn)*** *and* ***Interquartile Range (IQR)*** *alongside means to provide a more robust measure of central tendency.”*

*While Table 2 reports means and standard deviations for comparability with prior literature, we now also report: Overall Engagement Score Mdn = 42, IQR = [18, 89]. These values confirm that the distribution is indeed right-skewed, as expected.*

***Addressing Omitted Confounders:*** *We acknowledge that our regression model (Table 3) does not include several potentially relevant confounders, such as account verification status, bot likelihood, and time of posting. This omission was due to data availability constraints:*

- ***Account verification status****: Not reliably available in our dataset due to Twitter's policy changes during the study period.*
- ***Bot likelihood****: While we applied deduplication and spam filtering (as described in Section 3.1), we did not implement a dedicated bot classifier. However, we note that the exclusion of retweets from the engagement calculation mitigates the inflationary effect of bot-driven amplification.*
- ***Time of posting****: We did not control for circadian or weekly posting patterns, which could influence visibility and engagement.*

*We have added a note to Section 4.6 (Predictors of User Engagement) acknowledging these and it reads as follows: “Note: additional confounders, such as account verification status, bot likelihood, and time of posting which may influence engagement are not included due to data availability.”*

We believe these revisions have significantly strengthened the manuscript and look forward to your decision.

Sincerely,

The Authors of PONE-D-25-55931
