# Supplementary material for: Public sentiment and thematic evolution in the metaverse: A large-scale computational analysis of Twitter discourse
Source: PLoS One. 2026 May 13;21(5):e0345135. doi: 10.1371/journal.pone.0345135 (PMC13170826; doi:10.1371/journal.pone.0345135)
Supplement: Attachment — Submitted filename: Response to Editor.docx [file pone.0345135.s005.docx]

Response to Editor

Date: February 27, 2026

Dear Editor,

Thank you for your additional comments on our second revision of manuscript PONE-D-25-55931, "Public Sentiment and Thematic Evolution in the Metaverse: A Large-Scale Computational Analysis of Twitter Discourse" submit to PlosOne.

We appreciate the thoughtful and constructive feedback from the editor, and are grateful for the opportunity to address the concerns through this second round of revision. We have made changes to our paper in response to the editor’ feedback.

The attached memo describes these changes. To organise our memo, we assigned a progressive code number to each and every comment received (*in red italic*). As a result, we are now resubmitting a much improved and more compelling paper, and hope that you share our enthusiasm for the new draft. We look forward to hearing from you in due course.

Thanks for giving us an opportunity to contribute to PlosOne.

Sincerely,

The Authors of PONE-D-25-55931

Additional Editor Comments:

It has been noticed that several references were added during the revision process. Please add only relevant references (do not add references solely because they were mentioned by the reviewers).

*We are grateful for the opportunity to address the Editor’s concern through this second round of revision. We*  *would like to emphasise that all references were included based on our independent assessment of their relevance rather than in response to reviewers pressure, we have carefully reconsidered the added references added in the first review round and upon reflection, we agreed to remove few references from the manuscript, specifically References 6 and 7 (Buragohain et al., 2025a; 2025b). For this reason, the paragraph in the Introduction has been revised and References 6-7 were removed. The new paragraph in the Introduction reads as follows: "Yet despite this institutional investment, broader public adoption remains constrained by systemic security, privacy, and trust challenges — factors increasingly recognized as critical prerequisites for sustainable metaverse deployment [6]. Understanding how these structural barriers intersect with lived user experience, as expressed in organic public discourse, is therefore essential for developers, policymakers, and investors navigating this terrain."*

*While references 6-7 have been removed from the manuscript, reference 8, 43, 45, 46, and 50 are retained as they are substantively relevant and scientifically grounded our study. Specifically:*

1) *Vasudevan et al., 2025 (formerly reference 8 and now reference 6) is retained as it directly substantiates the security/privacy/trust framing. This reference was not suggested by any reviewer and maps directly onto the empirical themes identified in our data, particularly within the "General Discourse" macro-theme cluster (19,874 tweets), which prominently features privacy and policy concerns. Its inclusion reflects independent scholarly judgment, not reviewer compliance.*
2) *Van der Linden et al 2018; Thakur et al.2023; Fosch-Villaronga et al. 2021 (formerly 43–45; now references 41–43). These references form the basis of the substantially expanded Section 3.5 (Data Source Limitations), which was added wholesale in response to Reviewer 2's comment R2.4. That limitations section did not exist in the original submission and could not have carried citations. Van der Linden et al. supports our discussion of Twitter's structural bias toward polarized expression; Thakur et al. provides evidence for gender-based variation in metaverse perception; and Fosch-Villaronga et al. addresses regional adoption disparities. All three are required to give the limitation discussion scientific credibility rather than appearing as a pro forma acknowledgment.*
3) *Park & Kaye 2019; Boyd et al. 2010; Engelmann et al. 2019; Rehm et al. 2020; Zhang et al. 2024; (formerly 46–50; now references 44–48). These five references were added to address Reviewer 2's critique (R2.5) that our engagement metric weights (1.5× Retweets, 2× Replies, 3× Quote Tweets) lacked theoretical grounding. Without anchoring these weights in published literature on cognitive effort and network visibility, the metric design remained as the reviewer correctly noted, an unsupported arbitrary choice. These citations collectively provide the necessary empirical and theoretical basis for the weighting scheme and are integral to the methodological integrity of Section 3.4.*
